# Draft genomes of *Vibrio vulnificus* strains from the Albufera ecosystem in Valencia: isolates from summer 2022 to summer 2023

**DOI:** 10.1128/mra.00035-25

**Published:** 2025-03-27

**Authors:** Rubén Salvador-Clavell, Héctor Carmona-Salido, Carmen Amaro

**Affiliations:** 1 Department of Microbiology and Ecology and BIOTECMED Institute, University of Valencia 16781, Valencia, Spain; California State University San Marcos, San Marcos, California, USA

**Keywords:** *Vibrio vulnificus*, Albufera, Isolation, fish pathogens

## Abstract

Here, we report the genomes of 13 *Vibrio vulnificus* isolates from Albufera Lake, Valencia. The strains were isolated during the summers 2022 and 2023 when water temperature reached 30°C. This elevated water temperature is unusual in this ecosystem and may be influenced by global warming.

## ANNOUNCEMENT

*Vibrio vulnificus* (Vv) is a Gram-negative bacterium that inhabits brackish water in warm environments. However, due to climate change and increasing water temperatures, it has recently been isolated in colder waters, such as those of the Baltic Sea (1), as well as the Ebro Delta in the Mediterranean Sea (2). This pathogen can cause septicemia in fish and humans, known as vibriosis, posing both environmental and public health risks. Between 2022 and 2023, we performed four seasonal environmental water samplings in four locations within the Albufera Natural Park, situated south of Valencia, Spain: Albufera Lake, the two main boat channels connected to the sea (Pujol and Perellonet), and Perellonet Beach adjacent to the Perellonet boat channel. The collected water samples were cultured in *Vibrio vulnificus* medium (3), resulting in the isolation of 13 strains of Vv. The isolated bacteria were purified on Tryptic Soy Agar medium supplemented with 1% NaCl (TSA-1; Condalab, Spain) at 28°C for 24 h. These Vv isolates were confirmed through genetic analysis using the species-specific gene *vvhA* (4).

Next, the genomes of the Albufera isolates (Alb1, Alb2, Puj1, Puj2, Puj3, Puj4, Per1, Per2, Per3, Per4, Per5, Permar1, and Permar2) were sequenced. To achieve this, a single colony from a TSA-1 plate, incubated at 28°C for 24 h, was used to inoculate 5 mL of Lysogeny Broth with 1% NaCl (LB-1; Condalab, Spain) at 28°C for 24 h and 110 rpm. Genomic DNA was extracted using the MagAttract HMW DNA kit (Qiagen, Germany), following the manufacturer’s protocol for the “Purification of High-Molecular-Weight Genomic DNA From Gram-Negative Bacteria.” DNA integrity was verified by electrophoresis and Nanodrop analysis, and its concentration was quantified using an Invitrogen Qubit 3.0 fluorometer (Thermo Fisher Scientific, USA). Sequencing was performed using two techniques by the Central Support Service for Experimental Research (SCSIE) at the University of Valencia (Valencia, Spain). On one hand, the PacBio Sequel IIe platform (7- to 10-kb library) was used for Alb1, Per1, and Per2 strains. Libraries were prepared with the SMRTbell Express Template Preparation Kit v3.0, and sequencing was conducted according to the Sequel II sequencing kit 2.0 and SMRT cell 8M Tray protocols (approximately 10 kb for Per1 and 5 kb average read length for Alb1 and Per2). On the other hand, the library for Illumina sequencing was performed for the remaining isolates using the Nextera XT Library Preparation Kit. Illumina sequencing was carried out using MiSeq v3 2 × 300 bp for 600 cycles. Illumina reads were processed by SCSIE with Local Run Manager from Illumina with the Module GenerateFASTQ 3.0.1 trimming the adaptor "CTGTCTCTTATACACATCT" used in the library preparation. Read quality was analyzed using AfterQC v0.9.6, and filtering was applied by a quality threshold of 25. *De novo* genome assemblies were performed using Unicycler v0.5.0 (5) for Illumina reads or Flye v2.9-b1768 (6) for PacBio HiFi long-read data.

Table 1 summarizes the draft genome analysis of the Albufera isolates. The curated data sets were annotated using PGAP v6.7 for gene prediction (7). Finally, Quast v5.3.0 (8), using the total contigs function, was applied for genome analysis.

**TABLE 1:** Genome statistics for the Albufera strains isolated in a seasonal cycle of sampling from 2022 to 2023

Strain name	Season (Year)	Isolation site (coordinates)	Sequencing technique	Assembly method	Total genome size (bp)	No. of contigs	N50 (bp)	L50	Genome coverage (×)	GC content (%)	No. of genes	Genome accession no.	SRA no.	No. of reads
Alb1	Autumn (2022)	Albufera Lake (39.3492 N 0.3237 W)	PacBio	Flye	5,138,761	2	3,227,262	1	353	46.47	4,670	GCA_038397025.1	SRX27344741	307,142
Alb2	Spring (2023)	Albufera Lake (39.3492 N 0.3237 W)	Illumina MiSeq	Unicycler	5,201,471	181	145,973	13	127	46.53	4,826	GCA_037937605.1	SRX27344742	1,247,608
Puj1	Spring (2023)	Pujol boat channel (39.3484 N 0.3220 W)	Illumina MiSeq	Unicycler	4,853,448	57	280,612	6	141	46.73	4,400	GCA_037402835.1	SRX27333848	1,272,627
Puj2	Summer (2023)	Pujol b.ch. (39.3484 N 0.3220 W)	Illumina MiSeq	Unicycler	4,926,893	148	139,706	13	129	46.66	4,548	GCA_037402815.1	SRX27333849	1,269,631
Puj3	Summer (2023)	Pujol b.ch. (39.3484 N 0.3220 W)	Illumina MiSeq	Unicycler	4,946,707	121	235,823	7	117	46.62	4,516	GCA_037402755.1	SRX27333850	1,109,466
Puj4	Summer (2023)	Pujol b.ch. (39.3484 N 0.3220 W)	Illumina MiSeq	Unicycler	4,949,830	120	212,299	8	140	46.61	4,457	GCA_046720945.1	SRX27333851	1,306,218
Per1	Summer (2022)	Perellonet b.ch. (39.3084 N 0.2938 W)	PacBio	Flye	5,113,765	2	3,302,724	1	217	46.69	4,673	GCA_045348105.1	SRX27344743	109,331
Per2	Autumn (2023)	Perellonet b.ch. (39.3084 N 0.2938 W)	PacBio	Flye	5,078,888	3	3,291,740	1	355	46.68	4,698	GCA_038397015.1	SRX27344744	310,578
Per3	Spring (2023)	Perellonet b.ch (39.3084 N 0.2938 W).	Illumina MiSeq	Unicycler	4,864,978	224	199,461	9	151	46.65	4,462	GCA_037937235.1	SRX27344745	1,353,897
Per4	Spring (2023)	Perellonet b.ch. (39.3084 N 0.2938 W)	Illumina MiSeq	Unicycler	4,819,808	60	303,902	6	125	46.72	4,349	GCA_037937255.1	SRX27344746	1,092,144
Per5	Summer (2023)	Perellonet b.ch. (39.3084 N 0.2938 W)	Illumina MiSeq	Unicycler	4,966,847	103	152,830	10	123	46.64	4,548	GCA_037402695.1	SRX27333845	1,144,711
Permar1	Spring (2023)	Perellonet beach (39.3083 N 0.2915 W)	Illumina MiSeq	Unicycler	5,135,222	117	152,818	10	110	46.56	4,736	GCA_037402605.1	SRX27333846	1,094,962
Permar2	Summer (2023)	Perellonet beach (39.3083 N 0.2915 W)	Illumina MiSeq	Unicycler	4,913,732	129	350,736	6	93	46.71	4,572	GCA_037407335.1	SRX27333847	894,511

## Data Availability

This Whole Genome Shotgun project has been deposited in DDBJ/ENA/GenBank under the BioProject accession number PRJNA1087707 with genome accession number per strain (Table 1). The versions described in this paper are the first version for each strain.
